# Several proteins derived from serum exosomes are potential biomarkers for diagnosis and progression of sudden sensorineural hearing loss

**DOI:** 10.3389/fneur.2025.1700165

**Published:** 2025-11-12

**Authors:** Juanjuan Li, Suwen Bai, Peng Zhang, Xianhai Zeng, Hui Kong

**Affiliations:** 1 Departmemt of Otorhinolaryngology, The Second Hospital of Dalian Medical University, Dalian, China; 2 Department of Otolaryngology, Shenzhen Longgang Otolaryngology Hospital and Shenzhen Institute of Otolaryngology, Shenzhen, China; 3 SCUT-SLENT Digtal Hearing Healthcare Joint Lab, Shenzhen, China

**Keywords:** sudden sensorineural hearing loss, exosomes, biomarkers, different expression proteins, receiver operating characteristic curve

## Abstract

**Background:**

This study aims to compare the protein expression profiles of plasma-derived exosomes in patients with sudden sensorineural hearing loss (SSNHL) and normal hearing control groups to identify exosome proteins that may be associated with SSNHL or serve as biomarkers for SSNHL.

**Methods:**

Researchers collected peripheral venous blood from SSNHL patients and healthy controls for exosome isolation. The isolated exosomes were identified through nanoparticle tracking analysis, transmission electron microscopy observation, and Western blotting, followed by total protein extraction for proteomic sequencing. Differential expression proteins (DEPs) were screened using the threshold criteria of *p*-value<0.05 and fold change (FC) > 1.2, with subsequent functional analysis. Ultimately, four exosomal DEPs-RPS2, RPL19, ACO2, and APOE-were selected and validated using ELISA.

**Results:**

Researchers isolated exosomes from plasma and identified them through particle size analysis, morphological observation, and expression of exosome marker proteins. Comparative studies with healthy individuals revealed 363 DEPs in SSNHL. Additionally, 515 DEPs were identified in mild sudden deafness (MilSSNHL) and healthy controls, 982 in moderate cases (ModSSNHL) and healthy controls, and 1,161 in profound cases (ProSSNHL) and healthy controls. These proteins are involved in signaling pathways enriched by DEPs. Validation experiments demonstrated that the expression levels of these proteins consistently matched their sequencing results, ensuring high reliability. Furthermore, these candidate proteins show significant diagnostic potential for SSNHL.

**Conclusion:**

The four extracellular proteins identified in this study, including RPS2, RPL19, ACO2 and APOE, may be closely related to the occurrence and development of SSNHL or serve as biomarkers for the diagnosis and staging of SSNHL.

## 1 Introduction

Sudden sensorineural hearing loss (SSNHL) is generally defined as sensorineural hearing impairment of unknown etiology, characterized by a severe loss of at least 30 dB across at least three consecutive frequencies within 3 days (1). During the diagnostic process, the incidence of SSNHL is approximately 1.5–1.7 cases per 100,000 individuals (2–4). Fewer than half of the patients can have their etiology identified, allowing for specific treatment plans (2–5). For the majority of patients, the cause remains unknown. Despite extensive research, the treatment for patients with unknown etiology is still controversial. Regardless of the cause, the hearing threshold in SSNHL may either fail to recover, partially recover, or fully recover. Factors influencing hearing recovery include the age at the onset of hearing loss, the severity of the hearing loss and the affected frequencies, the presence of vertigo, and the time interval between the onset of hearing loss and the consultation with a doctor (2). Therefore, exploring the potential pathogenesis of SSNHL to formulate treatment plans and improve prognosis holds significant clinical importance.

Exosomes are extracellular vesicles surrounded by a lipid bilayer, which are released by most cell types. With a diameter of approximately 30–150 nm, they can mediate intercellular communication through receptor - ligand interactions or targeted delivery of substances (6). Breglio et al. (7) discovered that exosomes can prevent the death of cochlear hair cells induced by aminoglycoside antibiotics. Wong et al. (8) found the presence of exosomes in the inner ear and demonstrated that these exosomes have a protective effect against ototoxicity induced by cisplatin and gentamicin. Therefore, these results suggest the potential of exosomes as biomarkers (9, 10). However, there is limited research on the relationship between exosomes and SSNHL.

Exosomal proteins are either encapsulated within the membrane or embedded on its surface. As crucial components of exosomal vesicles, they reflect the physiological state of their parental cells and play significant roles in intercellular communication (11). In addition, compared with traditional tissue biopsy, plasma exosome protein has the characteristics of minimally invasive (only venous blood collection), stability (membrane protection) and timeliness (dynamic monitoring). Characterizing exosomal proteins provides deep insights into the properties of their originating cells, making them valuable tools for disease diagnosis, prognosis assessment, and therapeutic research.

In this investigation, we conducted a comparative analysis of serum-derived exosome protein expression profiles between patients experiencing SSNHL across the severity spectrum and a normal hearing control cohort. Our objective was to delineate distinct exosome proteins potentially implicated in SSNHL pathogenesis or capable of serving as biomarkers for the condition.

## 2 Methods and materials

### 2.1 Clinical samples

The studies involving human participants were reviewed and approved by the Medical Ethics Committees of Longgang Otorhinolaryngology Hospital (KY-2024-23-01). The participants provided their written informed consent to participate in this study. Inclusion criteria for specimen collection required that patients, newly diagnosed with SSNHL and no treatment (blood samples were taken immediately after admission), no previous trauma or surgery history, and no cranial nerve damage except for cranial nerve VIII. Exclusion criteria were as follows: diagnosis of herpes zoster infection, meniere’s disease, noise deafness, drug-induced ototoxicity, meningitis, vascular disease, metabolic disease, autoimmune disease and visceral diseases of other known etiology. Normal hearing controls were recruited among hospital staff, select the appropriate age and gender, and no underlying disease. Based on these criteria, patients with SSNHL and healthy volunteers were included in this study (Table 1).

**Table 1:** Physiological and biochemical indices of sudden sensorineural hearing loss (SSNHL) patients and healthy individuals.

Type	Number	Age	Height (cm)	Weight (kg)	Sex	Pure tone hearing, dBHL							
ProSSNHL							250 Hz	500 Hz	1,000 Hz	2000 Hz	4,000 Hz	8,000 Hz	Mean
	1	27	166	62	Male	Right	20	20	15	15	10	35	19.3
						Left	75	90	85	85	100	NR	90.8
	2	27	150	65	Female	Right	95	95	100	100	90	90	95
						Left	10	10	15	15	10	20	13.3
	3	30	159	52	Female	Right	80	105	90	115	NR	NR	101.7
						Left	10	10	10	15	15	10	11.7
	4	58	159	70	Male	Right	15	15	25	10	15	10	15
						Left	75	75	75	85	100	NR	86.7
ModSSNHL	1	35	159	62	Female	Right	5	5	5	5	5	5	5
						Left	45	45	55	55	60	60	53.3
	2	48	163	63	Female	Right	20	15	5	10	10	10	11.7
						Left	65	60	60	55	60	60	60
	3	35	182	82.7	Male	Right	45	50	65	45	50	55	51.7
						Left	5	10	5	5	20	5	8.3
	4	26	185	75	Male	Right	60	60	60	50	50	45	54.2
						Left	20	25	20	20	25	10	20
MilSSNHL	1	26	167	62.5	Male	Right	20	20	15	15	15	10	15.8
						Left	30	35	60	30	35	40	38.3
	2	37	164	58	Female	Right	10	15	10	5	15	10	10.8
						Left	50	45	40	25	25	20	34.2
	3	38	154	49.5	Female	Right	25	25	35	30	45	40	33.3
						Left	15	15	10	15	25	20	16.7
	4	51	175	73	Male	Right	10	10	10	20	10	20	13.3
						Left	35	25	35	40	40	40	35.8
Healthy	1	48	173	76	Male	Right	15	20	5	5	0	5	8.3
						Left	15	15	15	10	15	15	14.2
	2	49	175	66	Male	Right	10	15	15	20	10	10	13.3
						Left	5	0	5	10	15	10	7.5
	3	27	165	38.9	Female	Right	10	5	15	10	10	5	9.2
						Left	15	10	20	15	15	15	15
	4	32	163	47.9	Female	Right	15	20	10	15	10	5	12.5
						Left	5	0	0	5	10	5	4.2

### 2.2 Exosome isolation and identification

After venous blood collection in EDTA anticoagulant tube, it was immediately placed at 4 °C and centrifuged at 1,500 x g for 15 min to separate the plasma, and then packed into sterile enzyme-free cryopreservation tube and store at −80 °C. Each tube was packed with no more than 1 mL to avoid repeated freezing and thawing. Exosome isolation was carried out using the Total Exosome Isolation Reagent (Thermo Fisher Scientific, USA) according to the manufacturer’s instructions, followed by filtration through 0.22-μm polyethersulfone (PES) membrane filters. Exosome proteins concentration was determined using a Bicinchoninic acid (BCA) Protein Concentration Detection Kit (Beyotime Biotechnology, Shanghai, China). The exosomes were then added to the medium at different concentrations and incubated for 48 h (12).

### 2.3 Transmission electron microscopy (TEM)

Exosomes were fixed with overnight at 4 °C in 2.5% glutaraldehyde after washing in PBS, the cells were dehydrated, and sectioned (50–70 nm) using a Leica ultramicrotome (Leica, Germany). Sections were stained for 10 min with 2% uranyl acetate, followed by 5 min of lead staining Transmission electron microscopy (TEM) analysis was performed using a TEM (FEI, USA) at `120 kV. Images were captured via a CCD digital camera and analyzed using Soft Imaging (Olympus, Tokyo, Japan).

### 2.4 ELISA assays

Plasma-derived exosome were lysed by RIPA lysate (Beyotime Biotechnology, Shanghai, China). Then these proteins were determined using human RPS2, RPL19, ACO2 and APOE ELISA Kits (Animaluni, Shanghai, China) following the manufacturer’s instructions. The minimum significant level of detection was defined as 62.5 pg/mL for RPS2, 62.5 pg/mL for RPL19, 0.156 ng/mL for ACO2 and 3.12 ng/mL for APOE, as set by the manufacturer.

### 2.5 Protein–protein interaction (PPI) network analysis

PPI networks were analyzed using the Search Tool for the Retrieval of Interacting Genes (STRING; http://string-db.org) as described in a previous report (12). And the scores of related clusters and nodes were obtained by Cytoscape 3.5.1 software.

### 2.6 Statistical analysis

All experiments were performed with at least three biological replicates, and differences between the two groups of samples were analyzed using Student’s t-test. Statistical significance was set at a *p*-value of < 0.05.

## 3 Results

### 3.1 Identification of plasma-derived exosomes and their protein expression profiles in patients with healthy and SSNHL patients

**Figure 1:**
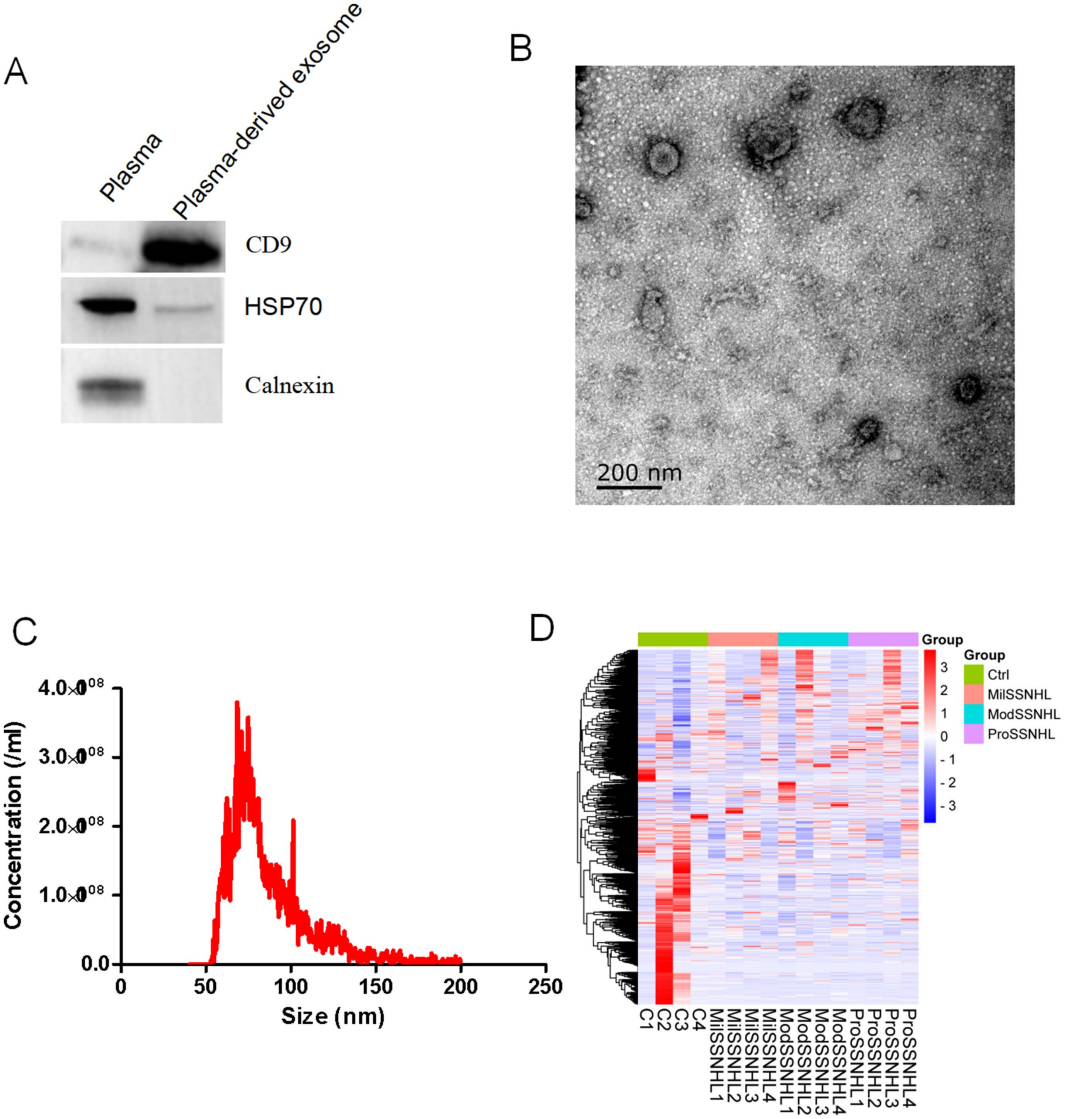
Identification of exosomes isolated from plasma and protein expression profiles in patients with healthy and SSNHL patients derived exosomes. **(A)** Western blotting showed that exosomal surface markers (CD9 and HSP70) were all expressed, and the endoplasmic reticulum signature protein (Calnexin) was not detected. **(B)** Transmission electron microscopy results show that exosomes have a double membrane structure and are disc-shaped. **(C)** Particle size distribution was measured by Nanosight. **(D)** Cluster analysis of exosomal proteins expression profiles. C1–C4, exosomes isolated from healthy individuals; MilSSNHL1-MilSSNHL4, exosomes isolated from mild sudden sensorineural hearing loss; ModSSNHL1-ModSSNHL4, exosomes isolated from moderate sudden sensorineural hearing loss; ProSSNHL, exosomes isolated from profound sudden sensorineural hearing loss.

To confirm that the vesicles we detected were exosomes, we first conducted an identification of exosomes (13). The results showed that the exosome marker proteins CD9, and HSP70 were expressed in the exosomes, while Calnexin was not expressed in the exosomes (Figure 1A). Electron microscopy results indicated that the vesicles we extracted exhibited a “saucer” shape (Figure 1B), and their particle sizes were mainly distributed around 75 nm (Figure 1C). Finally, the purity test showed that it was 4.5 × 10^−9^ μg/particles, indicating that the exosomes were not contaminated. Then, we analyzed plasma exosomes from each group (4 cases per group) based on LC/MS. The results showed that intra-group samples clustered closely, while inter-group samples clustered and separated significantly, indicating that SSNHL caused significant changes in plasma-derived exosomal proteins (Figure 1D). These results suggest that we successfully isolated plasma exosomes and the sequencing results were considered reliable.

### 3.2 Identification of a key protein in all stages of SSNHL

**Figure 2:**
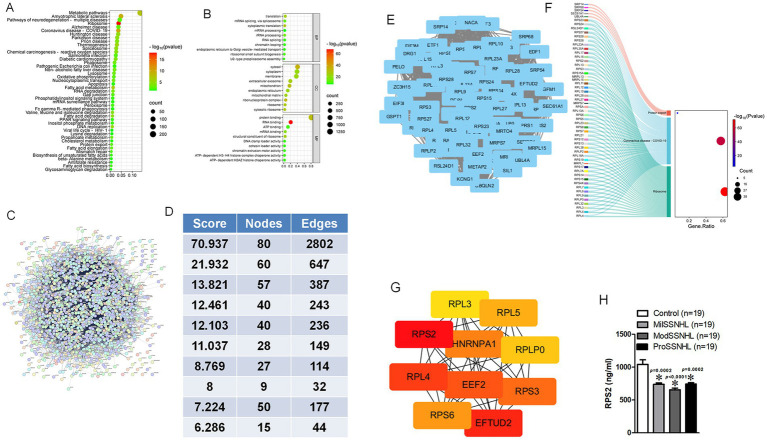
Exosomal RPS2 was significantly decreased in all stage of SSNHL patients. **(A)** Kyoto Encyclopedia of Genes and Genomes (KEGG) pathway enrichment scatterplot showing the all terms with the *p* < 0.05 via 363 DEPs. **(B)** Gene Ontology (GO) enrichment scatterplot showing the all terms with <0.05 via 363 DEPs. **(C)** Search Tool for the Retrieval of Interacting Genes (STRING) database showed the interaction of 363 DEPs. **(D)** Analysis of the top 10 clusters in the protein interaction network composed of 363 DEPs. **(E)** Interaction image of the protein interaction cluster with the highest score. **(F)** The participation of node signaling pathways in the cluster with the highest score in KEGG analysis. **(G)** The ranking analysis of all proteins involved in signaling pathways, with darker red scores higher. **(H)** The expression level of RPS2 was determined by ELISA in Control (healthy), MilSSNHL, ModSSNHL, and ProSSNHL groups, *n* = 19. Mean ± SE, *p < 0.05 compared to control using Student’s *t*-test.

A total of 363 proteins were identified as different expression proteins (DEPs) in SSNHL and healthy control samples based on the thresholds of FC > 1.2 and *p* < 0.05. KEGG pathway enrichment analysis showed that these DEPs were functionally enriched in the “Metabolic pathways,” “Ribosome,” “Pathways of neurodegeneration-mulitiple diseases” and “Oxidative phosphorylation” (Figure 2A). GO functional annotation analysis revealed that target genes of the identified DEPs were mainly related to “mRNA/rRNA processing,” “ribosome” and “RNA binding” (Figure 2B). STRING analysis revealed the interaction of DEPs, since protein interaction networks are usually highly complex, involving a large number of proteins and interactions, we performed STRING analysis on these DEPs, sorted the top 10 interaction clusters, and selected the highest ranked clusters for further analysis (Figures 2C–E). KEGG analysis of all proteins in this cluster showed that they were mainly enriched in “Protein export,” “Coronavirus disease-COVID-19” and “Ribosome” pathways (Figure 2F), after ranking the interacting proteins involved in these pathways, RPS2 was found to have the highest score (Figure 2G). The expression of RPS2 in SSNHL at different stages was verified by more samples. The results showed that the expression of RPS2 was significantly reduced in MilSSNHL, ModSSNHL, and ProSSNHL (Figure 2H). These results suggested that low expression of RPS2 is a key protein in all stages of SSNHL.

### 3.3 Identification of key proteins in different stages of SSNHL

**Figure 3:**
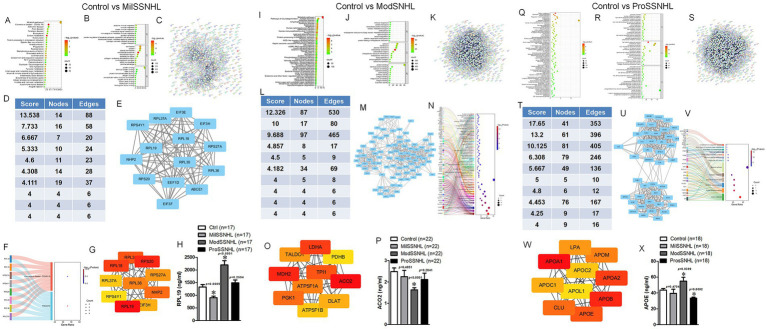
The plasma-derived exosomal RPL19, ACO2 and APOE are the key molecules in the MilSSNHL, ModSSNHL, and ProSSNHL stages, respectively. **(A)** KEGG pathway enrichment scatterplot showing the all terms with the *p* < 0.05 via 515 DEPs. **(B)** GO enrichment scatterplot showing the all terms with <0.05 via 515 DEPs. **(C)** STRING database showed the interaction of 515 DEPs. **(D)** Analysis of the top 10 clusters in the protein interaction network composed of 515 DEPs. **(E)** Interaction image of the protein interaction cluster with the highest score. **(F)** The participation of node signaling pathways in the cluster with the highest score in KEGG analysis. **(G)** The ranking analysis of all proteins involved in signaling pathways, with darker red scores higher. **(H)** The expression level of RPL19 was determined by ELISA in Control (healthy), MilSSNHL, ModSSNHL, and ProSSNHL groups, *n* = 17. **(I)** KEGG pathway enrichment scatterplot showing the all terms with the *p* < 0.05 via 982 DEPs. **(J)** GO enrichment scatterplot showing the all terms with <0.05 via 982 DEPs. **(K)** STRING database showed the interaction of 982 DEPs. **(L)** Analysis of the top 10 clusters in the protein interaction network composed of 982 DEPs. **(M)** Interaction image of the protein interaction cluster with the highest score. **(N)** The participation of node signaling pathways in the cluster with the highest score in KEGG analysis. **(O)** The ranking analysis of all proteins involved in signaling pathways, with darker red scores higher. **(P)** The expression level of ACO2 was determined by ELISA in Control (healthy), MilSSNHL, ModSSNHL, and ProSSNHL groups, *n* = 22. **(Q)** KEGG pathway enrichment scatterplot showing the all terms with the *p* < 0.05 via 1,161 DEPs. **(R)** GO enrichment scatterplot showing the all terms with <0.05 via 1,161 DEPs. **(S)** STRING database showed the interaction of 1,161 DEPs. **(T)** Analysis of the top 10 clusters in the protein interaction network composed of 1,161 DEPs. **(U)** Interaction image of the protein interaction cluster with the highest score. **(V)** The participation of node signaling pathways in the cluster with the highest score in KEGG analysis. **(W)** The ranking analysis of all proteins involved in signaling pathways, with darker red scores higher. **(X)** The expression level of APOE was determined by ELISA in Control (healthy), MilSSNHL, ModSSNHL, and ProSSNHL groups, *n* = 18. Mean ± SE, *p < 0.05 compared to control using Student’s *t*-test.

Using FC > 1.2 and *p* < 0.05 as thresholds, 515 DEPs were identified in mild sudden deafness (MilSSNHL) and healthy controls, 982 in moderate ((ModSSNHL)) and healthy controls, and 1,161 in profound (ProSSNHL) and healthy controls. KEGG pathway enrichment analysis showed that 515 DEPs were functionally enriched in the “Metabolic pathways,” “Endocytosis,” “Antigen processing and presentation” and “Phagosome” (Figure 3A). GO functional annotation analysis revealed that target genes of the 515 DEPs were mainly related to “immune response,” “extracellular exosome” and “protein/RNA binding” (Figure 3B). STRING analysis were performed and we sorted the top 10 interaction clusters, finally the highest ranked clusters was selected for further analysis (Figures 3C–E). KEGG analysis of these proteins, highest ranked cluster components, showed that they were mainly enriched in “Coronavirus disease-COVID-19” and “Ribosome” pathways (Figure 3F). RPL19 was founded to have the highest score after ranking the interacting proteins involved in these pathways (Figure 3G). The expression of RPL19 in SSNHL was identified by more samples. The results indicated that the expression of RPL19 was significantly reduced in MilSSNHL, but increased in ModSSNHL and have no change in ProSSNHL compared with health samples (Figure 3H). For ModSSNHL samples, we found that “Metabolic pathway,” “Protein processing in ER,” “cGMP-PKG signaling pathway” and “NOD-like receptor signaling pathway” were enriched in DEPs via KEGG analysis and GO analysis showed that “protein transport,” “extracellular exosome” and “protein binding” were enriched in these DEPs (Figures 3I,J). STRING interaction and screening score analysis revealed that ACO2 may be specifically expressed in the ModSSNHL samples (Figures 3K–O). Finally, the results showed that ACO2 was significantly low expressed in ModSSNHL, but there was no significant difference in its expression level in MilSSNHL and ProSSNHL (Figure 3P). For the ProSSNHL sample, we analyzed that DEPs were mainly involved in “Carbon metabolism,” “Endocytosis,” “HIF-1 signaling pathway” and “PI3K-Akt signaling pathway” (Figure 3Q). In addition, “glycolytic process,” “extracellular exosome” and “antioxidant activity” were enriched in ProSSNHL derived DEPs (Figure 3R). STRING interaction and screening score analysis revealed that APOE may be specifically expressed in the ProSSNHL samples (Figures 3S–W). The ELISA results showed that the expression level of APOE was significantly reduced in ProSSNHL samples, showed no significant change in MilSSNHL samples, and significantly increased in ModSSNHL samples (Figure 3X). These results suggest that the low expression of RPL19, ACO2 and APOE are key proteins in the plasma exosomes of MilSSNHL, ModSSNHL, and ProSSNHL patients, respectively.

### 3.4 Evaluation of diagnostic value of the obtained exosome-derived proteins via ROC curves

**Figure 4:**
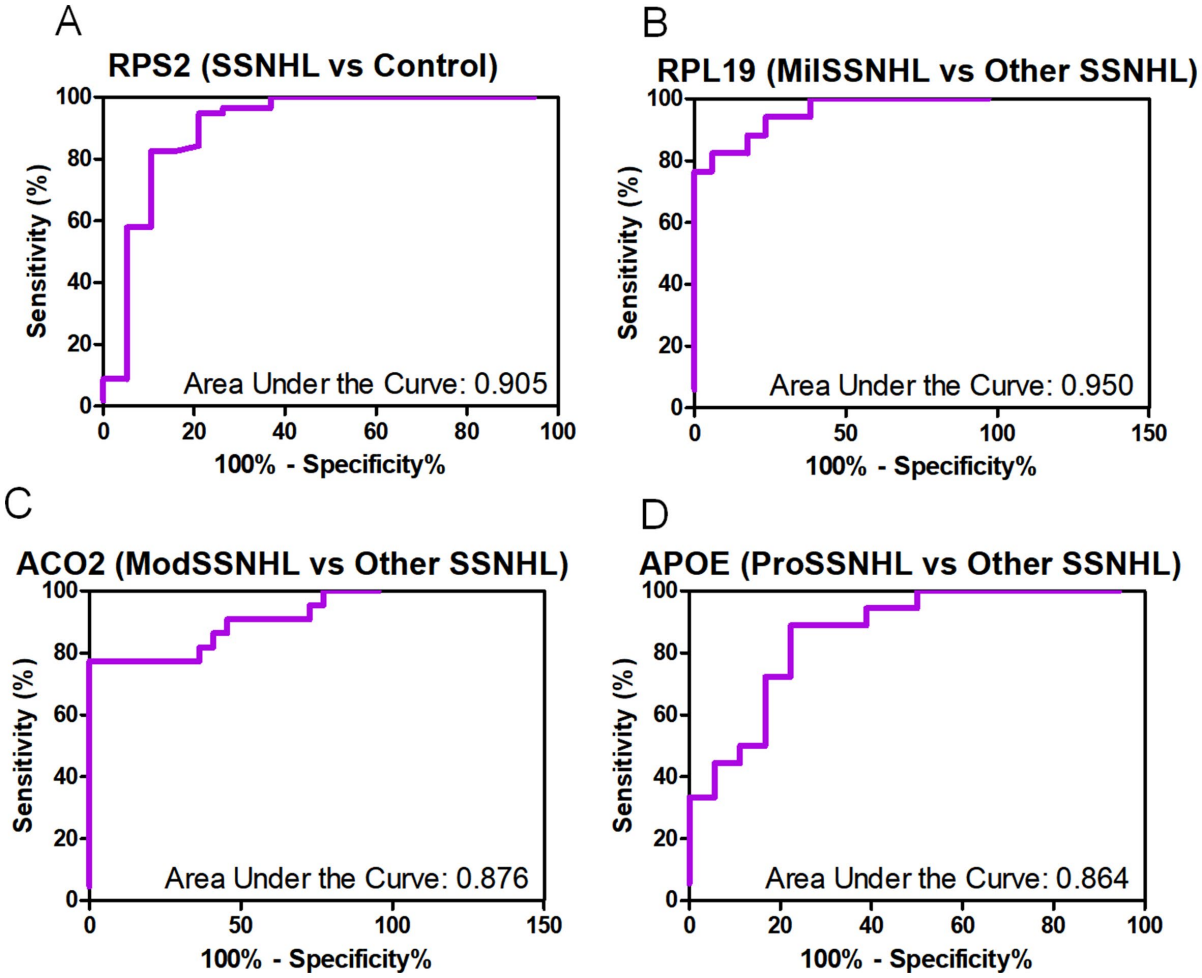
Diagnostic value of plasma-derived exosomal RPS2, RPL19, ACO2 and APOE in SSNHL patients. **(A)** Receiver operating characteristic curve analyses of RPS2 in SSNHL compared with healthy samples. **(B)** Receiver operating characteristic curve analyses of RPL19 in MilSSNHL compared with other SSNHL (ModSSNHL and ProSSNHL). **(C)** Receiver operating characteristic curve analyses of ACO2 in ModSSNHL compared with other SSNHL (MilSSNHL and ProSSNHL). **(D)** Receiver operating characteristic curve analyses of APOE in ProSSNHL compared with other SSNHL (MilSSNHL and ModSSNHL).

To evaluate the diagnostic value of plasma-derived exosomal RPS2 in SSNHL, we conducted a receiver operating characteristic curve (ROC) analysis comparing the diagnostic significance of RPS2 exosomes between SSNHL patients and healthy individuals. The results showed a strong diagnostic value (area under the curve = 0.905, 95% CI: 0.8046–1.006) that effectively distinguishes SSNHL patients (Figure 4A). To evaluate the diagnostic efficacy of plasma exosomal markers RPL19, ACO2, and APOE in different stages of SSNHL, we compared their ROC curves between MilSSNHL patients and other SSNHL samples (including ModSSNHL and ProSSNHL). The results demonstrated that plasma exosomal RPL19 exhibited exceptional discriminative power for MilSSNHL (area under the curve = 0.95, 95% CI: 0.8913–1.008) (Figure 4B), significantly outperforming other biomarkers. Additionally, ACO2 showed diagnostic value in distinguishing ModSSNHL samples (area under the curve = 0.876, 95% CI: 0.7664–0.9857) (Figure 4C), while APOE also demonstrated diagnostic significance in differentiating ProSSNHL samples (area under the curve = 0.864, 95% CI: 0.7453–0.9831) (Figure 4D). These results suggested that plasma-derived exosomal RPS2 has a good significance for the diagnosis of SSNHL, while plasma-derived exosomal RPL19 has a strong significance for the diagnosis of MilSSNHL, and plasma-derived exosomal ACO2 and APOE have a certain diagnostic value for the differentiation of ModSSNHL and ProSSNHL.

## 4 Discussion

SSNHL is a common emergency in otolaryngology. Early detection and timely intervention are crucial for improving hearing and alleviating tinnitus. Although pure-tone audiometry reveals diverse hearing curve patterns, systemic and intratympanic steroid therapy remains the primary treatment (14). Due to the lack of effective early diagnostic markers, this condition can only be confirmed through audiometric testing and medical history review after hearing loss occurs. Therefore, exploring potential biomarkers for sudden-onset sensorineural hearing loss holds significant research value.

A substantial body of scientific research has reported biomarker studies for SSNHL from multiple perspectives. Chen et al. identified thrombin time (TT) as a diagnostic biomarker for SSNHL and its predictive value in prognosis (15). Liu et al. (16) highlighted cerebellar signal abnormalities as neuroimaging biomarkers for SSNHL. Frosolini et al. (17) investigated the role of inflammatory biomarkers in SSNHL diagnosis and prognosis. Beyond imaging, serological, and plasma-based approaches, growing attention has been directed to exosome fluctuations and their functional roles in signaling transduction during inner ear pathologies (18). For example, Zhang et al. (19) suggested that plasma-derived exosomal Gm9866-miR-185-5p-Dusp7signaling pathway was identified to correlated with the occurrence and progression of immune-related hearing loss. Hao et al. (20) showed that nerual progenitor cells-derived exosomal miRNA-21 prevents hearing loss in mice caused by ischemia–reperfusion by inhibiting the inflammatory process of cochlea. Lai et al., reported that inner ear stem cells derived exosomal miR-182–5p can alleviate gentamicin induced ototoxicity, and enhance the survival capacity of HEI-OC1 cells (21). These studies highlight the potential value of exosomes as biomarkers for inner ear diseases. However, the complex structure of the inner ear within the temporal bone makes obtaining cochlear specimens extremely challenging (22, 23). Therefore, this study selected peripheral venous blood samples as an alternative source.

Exosomes exhibit significant potential as diagnostic biomarkers for various diseases due to their stable presence in body fluids and tissue-specific cargo. In non-cancer fields, exosomal markers have shown promise in diagnosing neurodegenerative disorders and acute organ injury (24–26), highlighting their broad applicability beyond oncology. However, research on exosomal DEPs in inner ear diseases—particularly SSNHL—remains markedly limited. Notably, the specific exosomal proteins regulating SSNHL pathogenesis and their stage-specific expression patterns remain unelucidated. This study addresses this gap by systematically profiling plasma-derived exosomal proteins and identifying stage-specific biomarkers for SSNHL, thereby advancing early detection and prognostic stratification of this debilitating condition. In this study, given the significant individual variations among samples and the small sample size (4 vs. 4), the statistical power of tests is compromised, making it particularly challenging to reveal true biological differences. Furthermore, the low abundance of exosome proteomes, their broad quantitative dynamic range, and the low expression levels of many functionally important proteins further complicate robust detection of differences. Therefore, we set the *p*-value threshold at <0.05 as the screening criterion (27, 28). However, this approach may increase the false positive rate. In the future study, we may apply FDR calibration for exosomal protein data, aiming to reduce false positives.

This study reveals that ribosomal protein RPS2, a key player in protein synthesis (29, 30), shows significantly low expression across all stages of SSNHL. And RPS2-mediated functions are closely related to 363 differentially expressed proteins (DEPs) that regulate ribosome activity, RNA binding, and protein output processes. In SSNHL patients, the lack of blood supply caused by endothelial dysfunction limits protein synthesis and secretion, which may be a possible factor in the decreased expression of RPS2 (30–33). This finding provides new insights for establishing RPS2 as a potential biomarker in SSNHL clinical diagnosis and treatment. Additionally, the analysis shows that the number of DEPs in MilSSNHL, ModSSNHL, and ProSSNHL subtypes increases stepwise with disease severity (515 → 982 → 1,161), indicating progressive proteomic dysregulation that may reflect cumulative cellular dysfunction from early metabolic/immune disturbances to advanced widespread signaling pathway destruction. Pathway enrichment analysis reveals distinct pathological features at each stage: MilSSNHL is marked by immune activation (antigen processing, phagocytosis) and metabolic imbalance; ModSSNHL manifest endoplasmic reticulum stress (protein processing) and NOD-like receptor-mediated inflammation; ProSSNHL involve hypoxia adaptation (HIF-1 pathway) and survival signals (PI3K-Akt). The dynamic expression pattern of RPL19 (MilSSNHL decreased, ModSSNHL increased, stable in ProSSNHL) suggests stage-dependent ribosome regulation mechanisms, potentially reflecting the body’s adaptive protein synthesis response to injury. The downregulation of subtype-specific candidate markers ACO2 and APOE correlates with energy metabolism disorders (tricarboxylic acid cycle) and lipid homeostasis imbalance associated with moderate-to-severe SSNHL, respectively. These findings highlight the potential value of RPL19/ACO2/APOE as a stage-specific therapeutic target and biomarker. However, further validation is required in larger sample cohorts with subgroup stratification (e.g., presence of vertigo or hearing recovery rate).

This study has several limitations. First, we only focused on validating the expression of DEPs with the highest scores, and have not yet conducted comprehensive testing on all DEPs. Second, the relatively limited sample size necessitates further experiments with larger sample sizes to validate the research conclusions. Finally, it remains unclear whether these biomarkers can effectively distinguish non-SSNHL patients. These issues will also be key focus areas for future research in this project.

## 5 Conclusion

To our knowledge, this study has for the first time constructed a differential expression proteome of plasma-derived exosomes in SSNHL patients, while analyzing and identifying candidate exosomal proteins with diagnostic value and potential regulatory mechanisms in SSNHL. These findings provide new insights for further exploration of SSNHL pathogenesis and related biomarkers.

## Data Availability

The datasets presented in this study can be found in online repositories. The names of the repository/repositories and accession number(s) can be found in the article/supplementary material.
